# Elevated CO_2_ alleviates the negative impact of heat stress on wheat physiology but not on grain yield

**DOI:** 10.1093/jxb/erz386

**Published:** 2019-08-24

**Authors:** Sachin G Chavan, Remko A Duursma, Michael Tausz, Oula Ghannoum

**Affiliations:** 1 ARC Centre of Excellence for Translational Photosynthesis, Hawkesbury Institute for the Environment, Western Sydney University, Penrith NSW Australia; 2 Department of Forest and Ecosystem Science, The University of Melbourne, Creswick, Vic., Australia; 3 The James Hutton Institute, UK

**Keywords:** Climate change, elevated CO_2_, grain yield, heat stress, photosynthetic acclimation, temperature response, wheat

## Abstract

Hot days are becoming hotter and more frequent, threatening wheat yields worldwide. Developing wheat varieties ready for future climates calls for improved understanding of how elevated CO_2_ (eCO_2_) and heat stress (HS) interactively impact wheat yields. We grew a modern, high-yielding wheat cultivar (Scout) at ambient CO_2_ (aCO_2_, 419 μl l ^−1^) or eCO_2_ (654 μl l^−1^) in a glasshouse maintained at 22/15 °C (day/night). Half of the plants were exposed to HS (40/24 °C) for 5 d at anthesis. In non-HS plants, eCO_2_ enhanced (+36%) CO_2_ assimilation rates (*A*_sat_) measured at growth CO_2_ despite down-regulation of photosynthetic capacity. HS reduced *A*_sat_ (–42%) in aCO_2_- but not in eCO_2_-grown plants because eCO_2_ protected photosynthesis by increasing ribulose bisphosphate regeneration capacity and reducing photochemical damage under HS. eCO_2_ stimulated biomass (+35%) of all plants and grain yield (+30%) of non-HS plants only. Plant biomass initially decreased following HS but recovered at maturity due to late tillering. HS equally reduced grain yield (–40%) in aCO_2_- and eCO_2_-grown plants due to grain abortion and reduced grain filling. While eCO_2_ mitigated the negative impacts of HS at anthesis on wheat photosynthesis and biomass, grain yield was reduced by HS in both CO_2_ treatments.

## Teaser

Elevated CO_2_ protects wheat photosynthesis from heat stress damage via increased electron transport and facilitates recovery of photosynthesis and biomass but not the yield due to heat-induced grain abortion.

## Introduction

Rising atmospheric CO_2_ concentration is the primary cause of increasing global mean surface temperatures as well as increased frequency, duration, and intensity of heat waves. Heat stress (HS), defined as short-term temperature increases above the optimum range (Wahid *et al.*, 2007), and other climate extremes such as droughts threaten global crop productivity, including wheat (Asseng *et al.*, 2015). Wheat (*Triticum aestivum* L.) is the most widely grown crop (>218 Mha planted annually) in the world, the second most produced cereal globally (771 Mt in 2017) after maize (1134 Mt in 2017) (FAO, 2019), and a significant source of protein, providing ~20% of global calories for human consumption. Recent trends in climate and global crop production (Lobell *et al.*, 2011) raise pertinent questions about the readiness of current crop genotypes to cope with future climate extremes, and highlight the need to evaluate the performance of current commercial, high-yielding crop genotypes under elevated CO_2_ (eCO_2_) and HS conditions.

Several studies have investigated the response of wheat to eCO_2_ (Kimball, 1983; Hocking and Meyer, 1991; Kimball et al., 1995, 1999; Nie *et al.*, 1995; Hunsaker et al., 1996, 2000; Miglietta *et al.*, 1996; Osborne *et al.*, 1998; Amthor, 2001). However, only a few studies have considered the interaction between eCO_2_ and warming in wheat (Rawson, 1992; Delgado *et al.*, 1994; Morison and Lawlor, 1999; Jauregui *et al.*, 2015; Cai *et al.*, 2016), and less frequently included HS (Wang et al., 2008, 2011; Shanmugam *et al.*, 2013; Fitzgerald *et al.*, 2016). Studies considering acute HS alone (Stone and Nicolas, 1994, 1996, 1998) or with eCO_2_ focused mainly on biomass or yield and not the underlying physiological processes such as photosynthesis. Only a few studies have considered the interactive effects of eCO_2_ and HS on wheat photosynthesis (Wang et al., 2008, 2011; Shanmugam *et al.*, 2013; Macabuhay *et al.*, 2018). These studies emphasize the need to determine the impacts of HS application at the vegetative and the important reproductive stage.

The FACE (free-air CO_2_ enrichment) study by Fitzgerald *et al.* (2016) in wheat relied on natural heat waves during the reproductive stage and highlighted the need for controlled-environment experiments in order to carefully investigate the interactive effects of eCO_2_ and HS on wheat productivity. The only FACE study with wheat by Macabuhay *et al.* (2018) involved controlled heat stress along with eCO_2_ and concluded that eCO_2_ may moderate some effects of HS on wheat grain yield, but such effects strongly depend on seasonal conditions and timing of HS. The limited number of studies highlight the gap in our understanding of how processes underlying wheat yield respond to the interactive effects of eCO_2_ and HS. Such understanding is important to identify potential adaptive traits for future breeding in order to stay abreast of climate change.

HS may cause irreversible effects on plant growth and development (Wahid *et al.*, 2007), and can inhibit both light and dark processes of photosynthesis via numerous mechanisms (Farooq *et al.*, 2011). For example, temperatures >45 °C can damage PSII (Berry and Bjorkman, 1980; Sage and Kubien, 2007). Plants may acclimate and acquire thermal tolerance to HS by activating stress response mechanisms and expressing heat shock proteins to repair HS damage (Pan *et al.*, 2018; Zhang *et al.*, 2018). Acquired thermotolerance is cost intensive and compromises plant productivity (Wahid *et al.*, 2007).

Elevated CO_2_ reduces stomatal conductance and increases photosynthesis rates by stimulating carboxylation and suppressing oxygenation of Rubisco known as photorespiration (Ainsworth and Rogers, 2007; Leakey *et al.*, 2009). Photosynthesis responds transiently to an instantaneous increase in temperature but may acclimate in response to long-term exposures (>1 d) to high temperature (Yamasaki *et al.*, 2002). Above the thermal optimum (*T*_opt_), high temperature reduces photosynthesis by increasing photorespiration and decreasing Rubisco activation (Eckardt and Portis, 1997). The maximal rate of RuBP carboxylation (*V*_cmax_) responds positively to temperatures as high as 40 °C, but the maximal rate of ribulose bisphosphate (RuBP) regeneration or electron transport (*J*_max_) generally decreases at lower temperatures in the range of 33 °C (Medlyn *et al.*, 2002). The relative effect of eCO_2_ on net photosynthesis is greater at high temperatures due to suppression of photorespiration (Long, 1991). Elevated CO_2_ may also increase the *T*_opt_ of photosynthesis (Borjigidai *et al.*, 2006; Alonso *et al.*, 2008; Ghannoum *et al.*, 2010). At eCO_2_, the response of photosynthesis to temperature becomes increasingly limited by *J*_max_ and Rubisco activation (Sage and Kubien, 2007). Therefore, the *T*_opt_ of photosynthesis will reflect that of *J*_max_ in plants grown at eCO_2_. Above *T*_opt_, acclimation of photosynthesis to high temperatures is associated with increased electron transport and/or heat stabilty of Rubisco activase (Sage and Kubien, 2007). Hence, even though *J*_max_ decreases with short-term increases in temperature, prolonged exposure to high temperaure may trigger photosynthetic acclimation and increase *J*_max_. Consequently, we predict that eCO_2_ will increase the *T*_opt_ of photosynthesis, and mitigate negative effects of HS on photosynthesis via increased electron transport (Hypothesis 1).

The effects of HS on plant biomass and grain yield depend on the magnitude and duration of HS. HS at the vegetative stage reduces biomass and grain yield mainly by speeding up plant development and reducing the time available to capture resources, and by reducing photosynthetic rates (Lobell and Gourdji, 2012). At the flowering or anthesis stage, HS reduces grain number due to pollen abortion, while at the grain-filling stage, HS reduces grain weight by limiting assimilate translocation and shortening the grain-filling duration (Wahid *et al.*, 2007; Farooq *et al.*, 2011; Prasad and Djanaguiraman, 2014). Elevated CO_2_ may alleviate the negative impact of HS on biomass and grain yield through stimulation of photosynthesis, improvement in plant water status due to reduced transpiration, and protection of the photosynthetic apparatus from HS damage. Furthermore, increased levels of sucrose and hexoses in plants grown at eCO_2_ are associated with increased spike biomass and fertile florets (Dreccer *et al.*, 2014) and osmotic adjustment (Wahid *et al.*, 2007) which can improve HS tolerance (Shanmugam *et al.*, 2013). Taken together, we hypothesize that HS applied at anthesis will negatively impact plant biomass and grain yield less in eCO_2_ than in ambient CO_2_ (aCO_2_) (Hypothesis 2).

The primary objective of this study was to test the performance of a current wheat champion genotype under future climate extremes. The chosen wheat cultivar, Scout, is a high yielding variety with very good grain quality and contains a putative high transpiration efficiency gene which can increase water use efficiency (https://www.pacificseeds.com.au/images/Icons/Products/Wheat/SNSWVICSA/ScoutVICSA.pdf). Considering the limitations of field conditions, we undertook our study under controlled environments to unravel the physiological underpinnings of the responses to eCO_2_ and HS. Consequently, we grew wheat (cultivar Scout) plants in a glasshouse at current aCO_2_ and future eCO_2_, and exposed half of the plants to a 5 d HS at 50% anthesis (Zadoks scale DC65). We investigated the interactive effects of eCO_2_ and HS on photosynthesis, biomass, and grain yield in Scout plants.

## Materials and methods

### Plant culture and treatments

The experiment was conducted in a glasshouse located at the Hawkesbury campus of Western Sydney University, Richmond, New South Wales. The commercial wheat cultivar Scout, which has a putative transpiration use efficiency gene (Condon *et al.*, 2004), was selected for the current experiment. Seeds were sterilized using 1.5% NaOCl_2_ for 1 min followed by incubation in the dark at 28 °C for 48 h in Petri plates. Sprouted seeds were planted in germination trays using seed raising and cutting mix (Scotts, Osmocote^®^) at ambient CO_2_ (419 μl l^−1^, day time average), temperature 22.3/14.8 °C (day/night average), relative humidity (RH; 62%, day time average), and natural light (see Supplementary Fig. S1a–e at *JXB* online). Day and night time averages were calculated from 10.00 h to 16.00 h and from 20.00 h to 06.00 h, respectively. Two-week-old seedlings were transplanted into individual cylindrical pots (15 cm diameter and 35 cm height) filled with sieved soil collected from the local site. Two glass house chambers were used for plant growth treatments, one with aCO_2_ and the other with eCO_2_. Each chamber had two bays with 50 plants in each bay. Fifty pots with one plant per pot were placed close to each other with a density of 24 plants m^–2^. At the transplanting stage, pots were randomly distributed into aCO_2_ and eCO_2_ chambers. Transplanted plants were grown under the current aCO_2_ (419 μl l^−1^, daytime average) and eCO_2_ (654 μl l^−1^, day time average) with 62 % (day time average) RH, 22.3/14.8 °C (day/night average) growth temperature, and natural light (800 μmol m^−2^ s^−1^, average daily maximum) (Supplementary Figs S1, S2). Half of the aCO_2_- and eCO_2_-grown plants were exposed to a 5 d HS treatment at 50% anthesis (13 weeks after planting, WAP). HS was applied by moving plants to a separate neighbouring chamber maintained at 40/24 °C (day/night average) air temperature and 71% (daytime average) RH during the 5 d HS treatment (Supplementary Figs S1a–e, S3). Plants were well watered throughout the experiment to separate HS and water stress effects. Thrive all-purpose fertilizer (Yates) was applied monthly throughout the experiment. To minimize chamber effects, pots were randomized regularly within and among the glasshouse chambers. Ten plants per treatment were used for physiological and biomass measurements.

### Temperature response of leaf gas exchange at five leaf temperatures

The response of the light-saturated CO_2_ assimilation rate (*A*_sat_) to variations in substomatal CO_2_ mole fraction (*C*_i_) was measured at five leaf temperatures (15, 20, 25, 30, and 35 °C) in both aCO_2_- and eCO_2_-grown plants before HS. Saturating light of 1800 μmol m^−2^ s^−1^ photosynthetic photon flux density (PPFD) was used for measurements. Six plants per treatment were used to measure one *A*–*C*_i_ curve (see below for details) at each temperature. Plants were transferred into a growth cabinet (Sanyo) with temperature and light control to achieve the desired leaf temperature by controlling air temperatures. Leaf temperature sequence started at 25 °C decreasing to 15 °C and then increased up to 35 °C. Dark respiration (*R*_d_) was measured by switching the light off for 20 min at the end of each temperature curve.

### Single leaf gas exchange measurements

Instantaneous steady-state leaf gas exchange measurements were performed before (9 WAP), during (13 WAP), after (13 WAP), and at the recovery stage (17 WAP) of the HS cycle using a portable open gas exchange system (LI-6400XT, LI-COR, Lincoln, NE, USA, equipped with a leaf fluorometer). Measurements were performed at a PPFD of 1800 μmol m^−2^ s^−1^ with two CO_2_ concentrations (400 μl l^−1^ and 650 μl l^−1^) and two leaf temperatures (25 °C and 35 °C). Measuring plants at common 25 °C gives an idea about photosynthetic acclimation, while measuring plants at common 35 °C indicates the effects of HS relative to control plants. Plants were moved to a neighbouring growth chamber to achieve the desired leaf temperature.

Parameters measured were light-saturated assimilation rate (*A*_sat_), stomatal conductance (*g*_*s*_), the ratio of intercellular to ambient CO_2_ (*C*_i_/*C*_a_), dark respiration (*R*_d_), and maximum light use efficiency of PSII of dark- and light-adapted leaves (*F*_v_/*F*_m_ and *F*_v_'/*F*_m_', respectively). These parameters were also measured in control plants before HS (13 WAP) and at the recovery stage (17 WAP) following HS. Photosynthetic down-regulation or acclimation was examined by comparing the measurements at common CO_2_ (aCO_2_- and eCO_2_-grown plants measured at 400 μl CO_2_ l^−1^) and growth CO_2_ (aCO_2_-grown plants measured at 400 μl CO_2_ l^−1^ and eCO_2_-grown plants measured at 650 μl CO_2_ l^−1^). *R*_d_ was measured after a 15–20 min dark adaptation period. Photosynthetic water use efficiency (PWUE), also termed intrinsic water use efficiency, was calculated as *A*_sat_ (μmol m^−2^ s^−1^)/*g*_s_ (mol m^−2^ s^−1^). The response of the *A*_sat_ to variations in *C*_i_ (*A*–*C*_i_ response curve) was measured at 17 WAP in eight steps of CO_2_ concentrations (50, 100, 230, 330, 420, 650, 1200. and 1800 μl l^−1^) at a leaf temperature of 25 °C. Measurements were taken around mid-day (from 10.00 h to 15.00 h) on attached fully expanded flag leaves (last leaves) of the main stems. Before each measurement, the leaf was allowed to stabilize for 10–20 min until it reached a steady state of CO_2_ uptake and stomatal conductance. Ten replicate plants per treatment were measured.

### Determination of Rubisco content 

Following gas exchange measurements, leaf discs (0.5 cm^2^) were collected using a cork borer from measured flag leaves, rapidly frozen in liquid nitrogen, and stored at –80 °C until analysed. Each leaf disc was extracted in 0.8 ml of ice-cold extraction buffer [50 mM EPPS–sodium hydroxide (pH 7.8), 5 mM DTT, 5 mM magnesium chloride, 1 mM EDTA, 10 µl of protease inhibitor cocktail (Sigma), and 1% (w/v) polyvinyl polypyrrolidone] using a 2 ml Tenbroeck glass homogenizer kept on ice. The extract was centrifuged at 15 000 rpm (21 130 rcf) for 1 min and the supernatant was used for the assay of Rubisco content. Samples were incubated in activation buffer [50 mM EPPS (pH 8.0), 10 mM MgCl_2_, 2 mM EDTA, 20 mM NaHCO_3_] for 15 min at room temperature. Rubisco content was estimated by the irreversible binding of [^14^C]CABP (2-C-carboxyarabinitol 1,5-bisphosphate) to the fully carbamylated enzyme (Sharwood *et al.*, 2008).

### Growth and biomass measurements

Plants were harvested at three time points: before HS (B), after recovery from HS (R), and at the final harvest after maturity (M). At each harvest, morphological parameters were measured and the biomass was harvested separately for roots, shoots, and leaves. Samples were dried for 48 h in the oven at 60 °C immediately after harvesting. Leaf area was measured before HS and at the recovery stage of HS using a leaf area meter (LI-3100A, LI-COR). Plant height, leaf number, tiller number, and spike (grain-bearing plant organ) number were also recorded. Leaf mass per area (LMA, g m^−2^) was calculated as total leaf dry mass/total leaf area.

### Mesophyll conductance and temperature response

Mesophyll conductance (*g*_m_) was determined by concurrent gas exchange and stable carbon isotope measurements using a portable gas exchange system (LI-6400-XT, LI-COR) connected to a tunable diode laser (TDL) (TGA100, Campbell Scientific, UT, USA) for Scout grown at ambient aCO_2_ partial pressures. *A*_sat_ and ^13^CO_2_/^12^CO_2_ carbon isotope discrimination were measured 35 d after planting at five leaf temperatures (15, 20, 25, 30, and 35 °C) and saturating light (1500 µmol quanta m^−2^ s^−1^). Leaf temperature sequence started at 25 °C decreasing to 15 °C and then increased up to 35 °C. Response of *A*_sat_ to variations in *C*_i_ was measured at each leaf temperature. R_*d*_ was measured by switching the light off for 20 min at the end of each temperature curve. Measurements were made inside a growth cabinet (Sanyo) to achieve the desired leaf temperature. The photosynthetic carbon isotope discrimination (Δ) to determine *g*_m_ was measured as follows (Evans *et al.*, 1986):

$$\begin{array}{l} \Delta_{}=\frac{1000\varepsilon (\delta {}^{13}C_{sam}-\delta {}^{13}C_{ref})}{1000+\delta {}^{13}C_{sam}-\varepsilon (\delta {}^{13}C_{sam}-\delta {}^{13}C_{ref})}. \end{array}$$ (1)

$$\begin{array}{l} Where,\varepsilon =\frac{C_{ref}}{C_{ref}-C_{sam}}. \end{array}$$ (2)

*C*_ref_ and *C*_sam_ are the CO_2_ concentrations of dry air entering and exiting the leaf chamber, respectively, measured by the TDL. *g*_m_ was calculated using correction for ternary and second-order effects (Farquhar and Cernusak, 2012; Evans and Von Caemmerer, 2013) following the next expression:

$$\begin{array}{l} g_{m}=\frac{\frac{1+t}{1-t}\left(b-a_{i}-\frac{eR_{d}}{A+R_{d}}\right)\frac{A}{C_{a}}}{\left(\Delta_{i}-\Delta_{o}-\Delta_{e}-\Delta_{f}\right)}. \end{array}$$ (3)

Where, Δ _i_ is the fractionation that would occur if the *g*_m_ were infinite in the absence of any respiratory fractionation (*e=*0), Δ _o_ is observed fractionation, and Δ _e_ and Δ _f_ are fractionation of ^13^C due to respiration and photorespiration, respectively (Evans and Von Caemmerer, 2013).

$$\begin{array}{l} \Delta_{i}=\frac{1}{1-t}a^{\prime }\frac{1}{\left(1-t\right)}((1+t)b-a^{\prime })\frac{C_{i}}{C_{a}}. \end{array}$$ (4)

$$\begin{array}{l} \Delta_{e}=\frac{1+t}{1-t}\left(\frac{eR_{d}}{(A+R_{d})C_{a}}(C_{i}-\Gamma *)\right). \end{array}$$ (5)

$$\begin{array}{l} \Delta_{f}=\frac{1+t}{1-t}\left(f\frac{\Gamma *}{C_{a}}\right). \end{array}$$ (6)

$$\begin{array}{l} Where,t=\frac{(1+a^{\prime })E}{2{g^{t}}_{ac}}. \end{array}$$ (7)

The constants used in the model were as follows: *E* denotes the transpiration rate; *g*^t^_ac_ is total conductance to diffusion in the boundary layer (*ab*=2.9‰) and in air (*a=*4.4‰); *a*′ is the combined fractionation of CO_2_ across the boundary layer and stomata; net fractionation caused by RuBP and phosphoenolpyruvate (PEP) carboxylation (*b=*27.3‰) (Evans *et al.*, 1986); fractionation with respect to the average CO_2_ composition associated with photorespiration (*f=*11.6‰) (Lanigan *et al.*, 2008); and we assumed null fractionation associated with mitochondrial respiration in light (*e=*0).

### Statistical analysis and curve fitting

The full factorial experimental design included measurement of 10 plants per treatment for gas exchange and biomass determination. Data analyses and plotting were performed using R computer software (R Core Team, 2017). The effect of treatments and their interactions were analysed using Student’s *t*-test and linear modelling with ANOVA. The homogeneity of variance was tested using Levene’s test from the car package. Significance tests were performed with ANOVA and post-hoc Tukey test using the ‘glht’ function in the multcomp package designed for multiple comparisons. Other packages were also used, including (but not limited to) lubridate (for effective use of dates in plots), sciplot (for plotting), doby (for calculating means and SEs), and visreg (for plotting). The significance levels for ANOVA were, *P<0.05, **P<0.01, and ***P<0.001. Coefficient means were ranked using post-hoc Tukey test.

The Farquhar–von Caemmerer–Berry (FvCB) photosynthesis model was fit to the A–*C*_i_ response curve or chloroplastic CO_2_ mole fraction (*C*_c_), which was estimated from the *g*_m_ measurements performed in a previous experiment as described above. *g*_m_ was measured in plants grown at aCO_2_ and assumed similar for plants grown at eCO_2_ due to the small effect of growth CO_2_ on *g*_m_ (Singsaas *et al.*, 2004). We employed the plantecophys R package (Duursma, 2015), which uses the FvCB model to perform fits using measured *g*_m_ and *R*_d_ values along with recently reported values for *K*_c_, energy of activation (*E*_a_) for *K*_c_, *K*_o_, and Γ* in wheat (Silva-Pérez *et al.*, 2017). Different temperatures values for *K*_c_, *K*_o_, and Γ*were determined using the Arrhenius equation as follows,

$$\begin{array}{l} f(Tk)=k_{25}\cdot \exp\left[\frac{E_{a}\cdot (Tk-298)}{R\cdot 298\cdot Tk}\right]. \end{array}$$ (8)

Where *E*_a_ is the activation energy (in J mol^−1^) and *k*_25_ is the value of the parameter at 25 °C. *R* is the universal gas constant (8.314 J mol^−1^ K^−1^), and *Tk* is the leaf temperature in K. The activation energy term *E*_a_ describes the exponential rate of rise of enzyme activity with the increase in temperature. Fitting the FvCB model using the plantecophys package resulted in estimates of maximal Rubisco carboxylation rate (*V*_cmax_) and maximal electron transport rate (*J*_max_). The temperature correction parameter (*T*_correct_) was set to False while fitting *A*–*C*_i_ curves using the plantecophys package. Means of coefficients were calculated using summaryBy function (in the doBy package). Means of estimated *V*_cmax_ and *J*_max_ values at five leaf temperatures were then fit by Arrhenius and peaked functions, respectively (Medlyn *et al.*, 2002), using the non-linear least square (nls) function in R to determine energy of activation for *V*_cmax_ (*E*_a_*V*) and *J*_max_ (*E*_a_*J*), and entropy (Δ*SJ*). Temperature responses of *V*_cmax_ and *R*_d_ means were fit using Arrhenius Equation 8. The temperature coefficient *Q*_10_, a measure of the rate of change of a parameter as a consequence of increasing the temperature by 10 °C, was also determined for *R*_d_ using the following equation:

$$\begin{array}{l} R_{d}=R_{d}25\cdot {Q_{10}}^{\left[(T-25)/10\right]} \end{array}$$ (9)

A peaked function (Harley *et al.*, 1992) derived Arrhenius function was used to fit the temperature dependence of *J*_max_, and is given by the following equation:

$$\begin{array}{l} f(Tk)=k_{25}\cdot \exp\left[\frac{H_{a}\cdot (Tk-298)}{R\cdot 298\cdot Tk}\right]\left[\frac{1+\exp\left(\frac{298\cdot \Delta S-H_{d}}{298\cdot R}\right)}{1+\exp\left(\frac{Tk\cdot \Delta S-H_{d}}{Tk\cdot R}\right)}\right] \end{array}$$ (10)

Where *H*_a_ is the activation energy and *k*_25_ is the *J*_max_ value at 25 °C, *H*_d_ is the deactivation energy, and *S* is the entropy term. *H*_d_ and Δ*S* together describe the rate of decrease in the function above the optimum. *H*_d_ was set to constant 200 kJ mol^−1^ to avoid overparameterization. The temperature optimum of *J*_max_ was derived from Equation 2 (Medlyn *et al.*, 2002) and written as follows:

$$\begin{array}{l} T_{opt}=\frac{H_{d}}{\Delta S-R\cdot \ln\left[\frac{E_{a}}{\left(H_{d}-E_{a}\right)}\right]} \end{array}$$ (11)

The temperature response of *A*_sat_ was fit using a simple parabola equation (Crous *et al.*, 2013) to determine the temperature optimum of photosynthesis:

$$\begin{array}{l} A_{sat}=A_{opt}-b\cdot {\left(T-T_{opt}\right)}^{2} \end{array}$$ (12)

where *T* is leaf temperature during measurement of *A*_sat_, *T*_opt_ represents the temperature optimum, and *A*_opt_ is the corresponding *A*_sat_ at *T*_opt_. Steady-state gas exchange parameters *g*_m_, *g*_s_, *C*_i_, and the *J*_max_ to *V*_cmax_ ratio were fit using the nls function with the polynomial equation:

$$\begin{array}{l} y+A+Bx+Cx^{2} \end{array}$$ (13)

## Results

### In non-HS plants, photosynthetic acclimation to eCO_2_ was stronger at the vegetative stage, while eCO_2_ stimulated photosynthesis at 25 °C at all growth stages

**Fig. 1.**
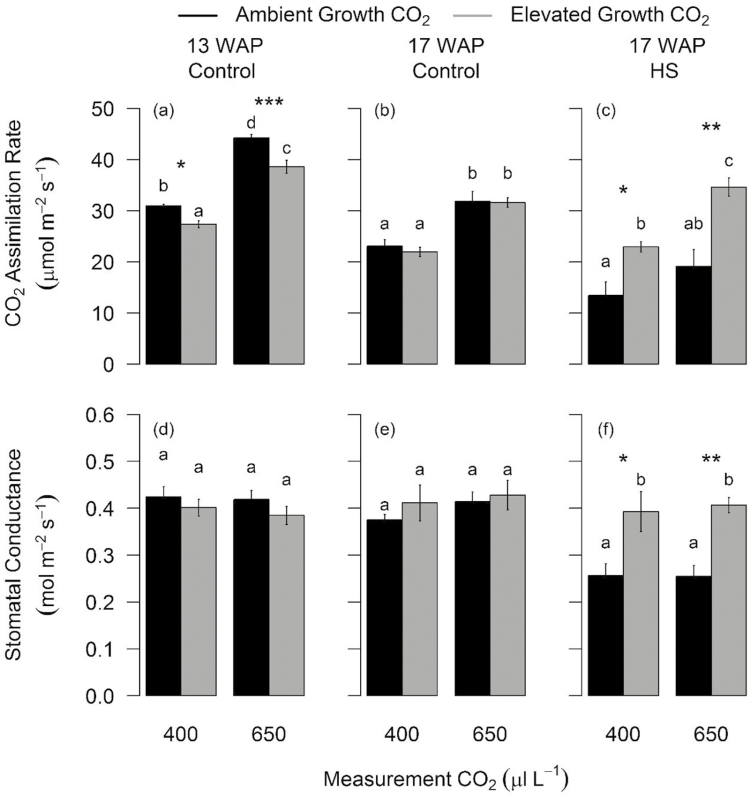
Photosynthetic response of wheat cultivar Scout to eCO_2_ measured 13 and 17 weeks after planting (WAP) at 25 °C leaf temperature and two CO_2_ concentrations. Bar plot of means for light-saturated CO_2_ assimilation rate (a, b, and c) and stomatal conductance (d, e, and f) calculated using two-way ANOVA. The error bars indicate the SE of the mean (*n*=9–10). Ambient and elevated CO_2_-grown plants are depicted in black and grey, respectively. Grouping is based on measurement CO_2_ (400 μl l^−1^ or 650 μl l^−1^). Bars sharing the same letter in the individual panels are not significantly different according to Tukey’s HSD test at the 5% level. Statistical significance levels (*t*-test) for eCO_2_ effect are shown: *P<0.05; **P<0.01: ***P<0.001.

To assess photosynthetic acclimation due to eCO_2_, non-HS plants were measured at the peak growth period (13 WAP) and after 50% anthesis (17 WAP). At 13 WAP, growth under eCO_2_ reduced *A*_sat_ measured at common CO_2_ at both 25 °C (–12%, *P*=0.004) (Fig. 1a; Table 1; and Supplementary Table S1) and 35 °C (–13.3%, *P*=0.01) (Table 1; Supplementary Table S1). At 13 WAP, eCO_2_ enhanced *A*_sat_ of non-HS plants measured at growth CO_2_, at both 25 °C (+25%, *P*=0.003) (Fig. 1a; Table 1; Supplementary Table S1) and 35 °C (+39%, *P*<0.001) (Table 1; Supplementary Table S1).

**Table 1.** Summary of statistics for gas exchange parameters

Parameter (mean plant^−1^)	Measurement		13 WAP	17 WAP		
	Temperature °C	CO_2_ (μl l^−1^)	Main effects	Main effects		Interaction
			CO_2_	CO_2_	HS	CO_2_×HS
*A* (µmol m^−2^ s^−1^)	25	400	**	*	*	**
		650	**	**	*	**
	35	400	**	NS	NS	*
		650	**	NS	NS	*
*R*_d_ (µmol m^−2^ s^−1^)	25	400	NS	NS	NS	NS
	35	400	*			
*g*_s_ (mol m^−2^ s^−1^)	25	400	NS	*	*	NS
		650	NS	**	**	*
	35	400	NS	NS	NS	NS
		650	NS	NS	NS	NS
PWUE (*A*/*g*_s_)	25	400	NS	NS	NS	NS
		650	NS	NS	NS	NS
	35	400	NS	NS	*	*
		650	NS	NS	NS	NS
*F*_v_/*F*_m_	25	400	NS	*	NS	NS
*F*_v_'/*F*_m_'	25	400	NS	**	**	*
*A* (µmol m^−2^ s^−1^)	25	Growth CO_2_	**	***	NS	**
	35		***	**	NS	*
*R*_d_ (µmol m^−2^s^−1^)	25		***			
	35		*			
*g*_s_ (mol m^−2^ s^−1^)	25		NS	***	**	*
	35		NS	NS	NS	NS
PWUE (*A*/*g*_s_)	25		***	***	NS	*
	35		**	***	NS	NS

Summary of statistical analysis using two-way ANOVA for the effects of elevated CO_2_ and heat stress (HS) on leaf gas exchange parameters measured at 13 and 17 weeks after planting (WAP). HS plants were measured at the recovery stage (*n*=9–10). Significance levels are **P<0.001; ** *P* <0.01; * *P* <0.05; NS, *P*>0.05.

Relative to 13 WAP, *A*_sat_ decreased after 50% anthesis (17 WAP), but was not affected by eCO_2_ in non-HS plants measured at common CO_2_ and 25 °C or 35 °C (Fig. 1b, c; Table 1; Supplementary Table S1). When non-HS plants were measured during anthesis at growth CO_2_, eCO_2_ increased *A*_sat_ at 25 °C (+36%, *P*<0.001) but not at 35 °C (Fig. 1b , c; Table 1; Supplementary Table S1). eCO_2_ had no significant effect on *g*_s_ of non-HS plants (Morison, 1998) measured 13 or 17 WAP at common or growth CO_2_ (Fig. 1d–f; Table 1; Supplementary Table S1).

In non-HS plants, thermal responses of leaf gas exchange differed between aCO_2_ and eCO_2_ at higher temperatures

**Fig. 2.**
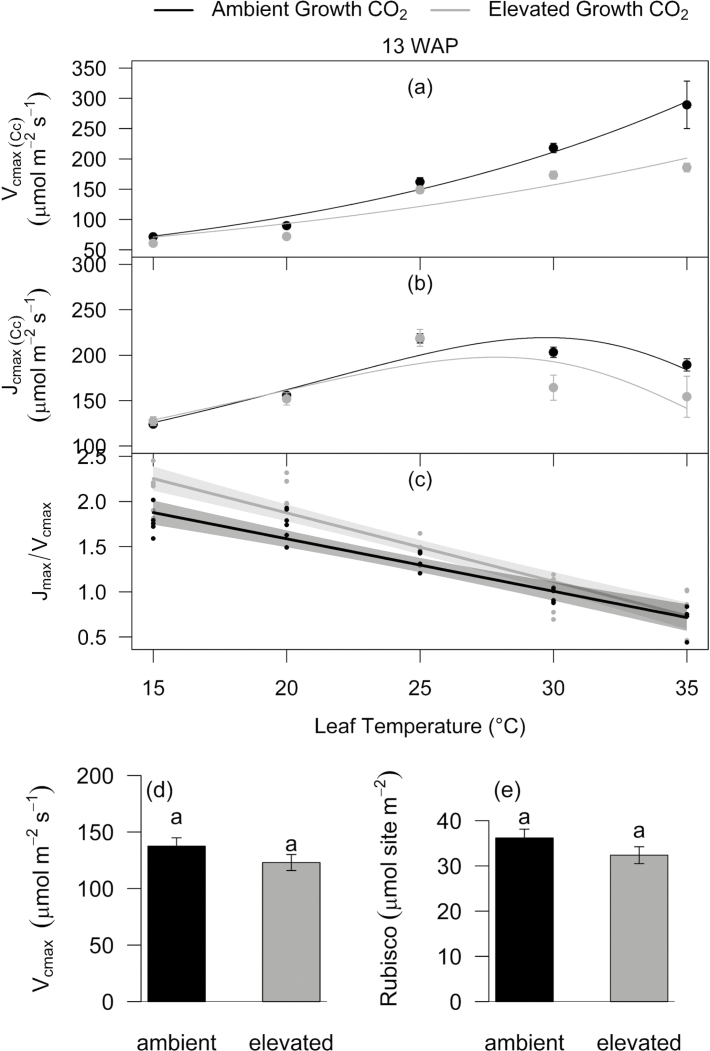
*In vivo* Rubisco properties and temperature response of *V*_cmax_ and *J*_max_ measured 13 weeks after planting (WAP). Maximum velocity of carboxylation, *V*_cmax_ (a), maximum velocity of RuBP regeneration, *J*_max_ (b), and *J*_max_*/V*_cmax_ ratio (c) determined using the response of CO_2_ assimilation to variation in chloroplastic CO_2_ (*C*_c_) at five leaf temperatures (15, 20, 25, 30, and 35 °C) in wheat cultivar Scout (*n*=6). The ratio of *J*_max_*/V*_cmax_ (c) is plotted using the visreg package in R. Regression lines are means with 95% confidence intervals. The lower panel is a bar plot showing *in vivo V*_cmax_ at 25 °C (*n*=6) (d) and Rubisco sites (*n*=5) (e) measured in flag leaf discs harvested at the same time point. For (a), (c), and (d), values are means ±SE. Ambient and elevated CO_2_-grown plants are shown in black and grey, respectively.

*A*–*C*_i_ curves were measured at five leaf temperatures to characterize the thermal photosynthetic responses of wheat plants grown at aCO_2_ and eCO_2_ (Fig. 2; Table 2; Supplementary Fig. S4). In non-HS, aCO_2_- and eCO_2_-grown Scout, *A*_sat_, *g*_s_, and *C*_i_ increased with temperature up to *T*_opt_ of ~23.5 °C and decreased more under eCO_2_ relative to aCO_2_ at higher temperatures. Relative to aCO_2_, plants grown under eCO_2_ had higher *A*_sat_ up to *T*_opt_ but similar *A*_sat_ at higher temperatures (Supplementary Fig. S4a). *R*_d_ increased with temperature under both aCO_2_ and eCO_2_; however, the rate of increase was slower at higher temperatures under eCO_2_, resulting in lower *R*_d_ under eCO_2_ relative to aCO_2_ at 30°C and 35 °C. Nevertheless, energy of activation (*E*_a_*R*) and the *Q*_10_ coefficient (rate of change due to an increase of temperature by 10 °C) of *R*_d_ were similar under aCO_2_ and eCO_2_ (Table 2; Supplementary Fig. S4b).

**Table 2.** Summary of modelled parameters for temperature response of photosynthesis

Parameter	Constant	Ambient growth CO_2_	Elevated growth CO_2_
*A*_sat_ (µmol m^−2^ s^−1^)	*T*_opt_ (°C)	23.7±1.1 a	23.4±1.3 a
	*A*_opt_	25.5±1.3 a	30.9±2.7 b
*V*_cmax_ (µmol m^−2^ s^−1^)	*V*_cmax_ at 25 °C	149±6 a	121±12 a
	*E*_a_ *V* (kJ mol^−1^)	51±4 a	38±10 a
*J*_max_ (µmol m^−2^ s^−1^)	*J*_max_ at 25 °C	200±12 a	190±22 a
	*T*_opt_ (°C)	29.5±0.7 a	27.5±0.9 a
	*J*_max_ at *T*_opt_	233±6	210±11 a
	*E*_a_ *J* (kJ mol^−1^)	37±11 a	34±22 a
	Δ*SJ* (J mol^−1^ K^−1^)	648±5 a	651±8 a
	*H*_d_ (kJ mol^−1^)	200	
*R*_d_ (µmol m^−2^ s^−1^)	*R*_d_ at 25 °C	2.4±0.1 a	2.2±0.1 a
	*E*_a_ *R* (kJ mol^−1^)	41±3 a	31±6 a
	*Q*_10_	1.73±0.07 a	1.50±0.13 a

Summary of coefficients derived using non-linear least square fitting of CO_2_ assimilation rates, maximal rate of carboxylation (*V*_cmax_), and maximal rate of RuBP regeneration (*J*_max_) determined using *A*–*C*_i_ response curves and dark respiration measured at five leaf temperatures (15, 20, 25, 30, and 35 °C). Values are means ±SEs. Derived parameters include temperature optima (*T*_opt_) of photosynthesis (*A*_opt_); activation energy for carboxylation (*E*_a_*V*); activation energy (*E*_a_*J*)¸ entropy term (∆*SJ*), and *T*_opt_ and corresponding value for *J*_max_ with deactivation energy (*H*_d_) assumed constant; and activation energy (*E*_a_*R*) and temperature coefficient (*Q*_10_) for dark respiration. Letters indicate significance of variation in means (*n*=6)

*V*_cmax_ and *J*_max_ were calculated by fitting the response of *A*_sat_ to variations in *C*_c_ (*A*–*C*_c_ response curve) using measured *R*_d_ and *g*_m_. *g*_m_ increased up to 25 °C and remained relatively unchanged at higher temperatures (Supplementary Table S3). Within the range of measured leaf temperatures, *V*_cmax_ increased with leaf temperature, while *J*_max_ increased up to a *T*_opt_ of 28 °C and decreased thereafter. *V*_cmax_ and *J*_max_ decreased with eCO_2_ at the two highest temperatures (Fig. 2). The *J*_max_/*V*_cmax_ ratio was higher under eCO_2_ relative to aCO_2_ at lower temperatures and decreased with leaf temperature under aCO_2_ or eCO_2_ (eventually being similar at 35 °C) (Fig. 2). Despite variations in the temperature response, the overall fitted parameters were mostly similar in plants grown at aCO_2_ or eCO_2_, except for *A*_opt_ which was higher under eCO_2_ (Table 2). There was no significant difference in *V*_cmax_ at 25 °C, *J*_max_ at 25 °C, *in vitro* measured Rubisco sites, or their activation energy under aCO_2_ or eCO_2_ (Fig. 2; Table 2).

### Photosynthesis and PSII efficiency decreased during HS at both CO_2_ treatments but recovered only under eCO_2_

In this study, we successfully implemented a 5 d HS cycle at 50% anthesis as evidenced by the higher leaf temperature of the HS relative to the control plants (Supplementary Fig. S1f). Overall, HS reduced photosynthesis and was more damaging in aCO_2_ than in eCO_2_ plants (Fig. 4; Supplementary Table S1). Before HS (15 WAP), eCO_2_ increased both *A*_sat_ (+43%, *P*<0.001) and *g*_s_ (+20%, *P*=0.032) measured at growth CO_2_. HS reduced *A*_sat_ measured during and after HS in both CO_2_ treatments. HS increased *g*_s_ measured during HS and reduced *g*_s_ after HS. One week after HS, both *A*_sat_ and *g*_s_ had completely recovered in eCO_2_-grown plants but not in aCO_2_-grown plants, which showed significant reductions in *A*_sat_ (–42%, *P*=0.017) and *g*_s_ (–32%, *P*=0.006) (Fig. 4a, c; Table 1; Supplementary Table S1).

**Fig. 3.**
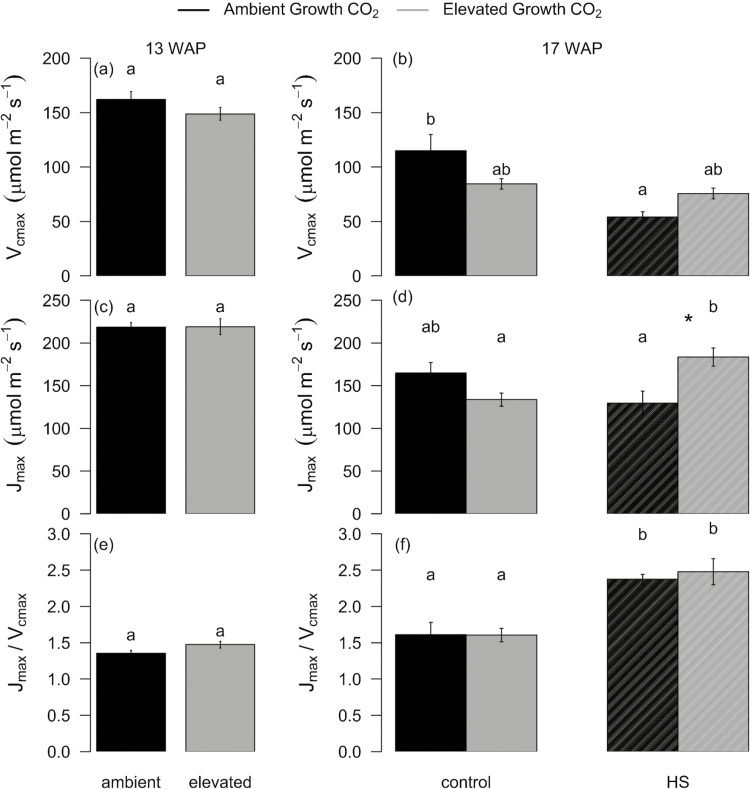
Response of *V*_cmax_ and *J*_max_ to growth at eCO_2_ and heat stress (HS) measured 13 and 17 weeks after planting (WAP) at the recovery stage of the HS cycle. Bar plot of means ±SE for *V*_cmax_ (a and b), *J*_max_ (c and d), and *V*_cmax_/*J*_max_ (e and f) using two-way ANOVA. Leaf gas exchange was measured at 25 °C in ambient (black) and elevated (grey) CO_2_-grown plants exposed (HS) or not exposed (Control) to a 5 d HS. Bars sharing the same letter in the individual panels are not significantly different according to Tukey’s HSD test at the 5% level. The error bars indicate the SE of the mean (*n*=9–10). Statistical significance levels (*t*-test) for eCO_2_ effect are shown: *P<0.05; **P<0.01: ***P<0.001.

The lasting negative effect of HS on photosynthesis at the recovery stage was associated with reduced *V*_cmax_ (–53%, *P*=0.002) in aCO_2_- but not in eCO_2_-grown plants. Conversely, the photosynthetic recovery after HS was associated with increased *J*_max_ (+37%, *P*=0.001) in eCO_2_- but not in aCO_2_-grown plants (Fig. 3d). Interestingly, HS significantly increased the *J*_max_/*V*_cmax_ ratio in both aCO_2_- and eCO_2_-grown plants, but the ratio was not affected by growth CO_2_.

**Fig. 4.**
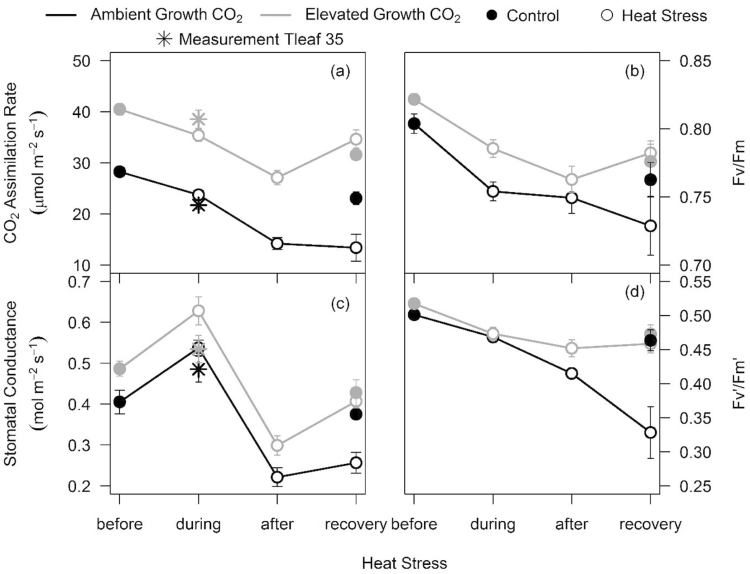
Photosynthesis and chlorophyll fluorescence response of aCO_2_- and eCO_2_-grown wheat cv. Scout measured before, during, after, and at the recovery stage of the heat stress cycle. CO_2_ assimilation rates (a), of the *F*_v_/*F*_m_ ratio in dark-adapted leaves (b), stomatal conductance (c), and of the *F*_v_'/*F*_m_' ratio in light-adapted leaves (d) measured at growth CO_2_ (aCO_2_-grown plants measured at 400 μl l^−1^ and eCO_2_-grown plants measured at 650 μl l^−1^). Values are means ±SE (*n*=9–10). Ambient and elevated CO_2_-grown plants are depicted in black and grey, respectively. Filled and open circles represent control and heat-stressed plants, respectively. The circle and star symbols depict CO_2_ assimilation rates measured at 25 °C and 35 °C, respectively.

Chlorophyll fluorescence measurements confirmed the persistent HS damage to photosynthesis in aCO_2_- relative to eCO_2_-grown plants. HS reduced light-adapted *F*_v_'/*F*_m_' measured after and at the recovery stage of HS in aCO_2_- (–29%, *P*=0.019) but not in eCO_2_-grown plants (Fig. 4d). HS reduced dark-adapted *F*_v_/*F*_m_ in aCO_2_- more than in eCO_2_-grown plants; and *F*_v_/*F*_m_ failed to recover in aCO_2_ plants after HS (Fig. 4b).

### At maturity, total plant biomass, but not grain yield, recovered from HS in both CO_2_ treatments

**Fig. 5.**
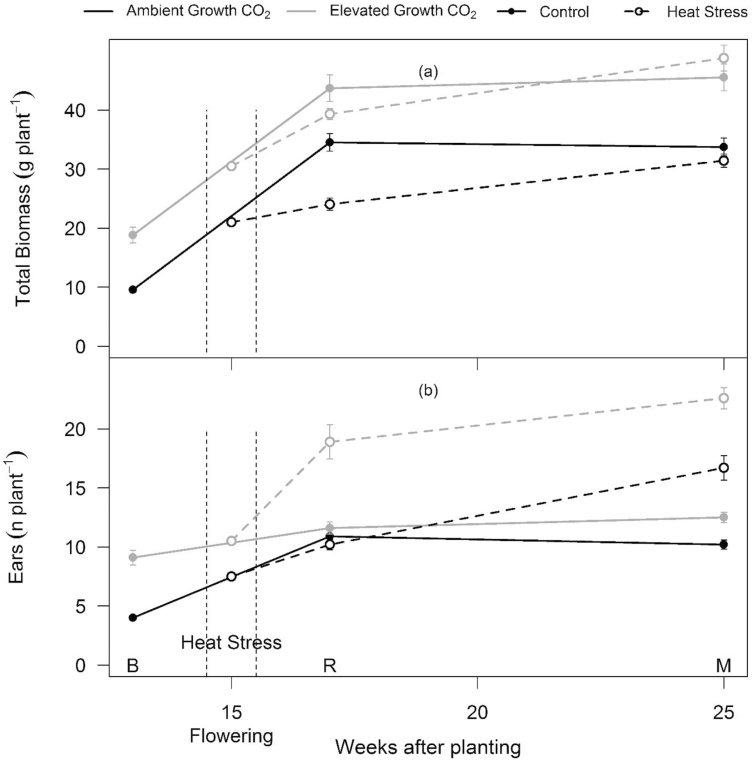
Response of biomass and ears (or tillers) to eCO_2_ and HS across the life cycle of wheat cv. Scout. Response of total biomass (a) and spike number (b) to eCO_2_ and HS at three time points; before HS (B), after recovery from HS (R), and at the final harvest after maturity (M). Ambient and elevated CO_2_-grown plants are depicted in black and grey, respectively. Solid and dotted lines represent control and heat-stressed plants, respectively. Filled and open circles represent control and heat-stressed plants, respectively. Vertical black dotted lines show the timing of HS. Symbols are means per plant ±SE (*n*=9–10).

Elevated CO_2_ stimulated growth rate, biomass, and grain yield. A faster growth rate was evident from the larger number of ears (+127%, *P*<0.001) in eCO_2_- relative to aCO_2_-grown plants harvested 13 WAP (before HS) (Fig. 5). Elevated CO_2_ significantly stimulated the total biomass harvested throughout the growing period (Fig. 5; Table 3; Supplementary Table S2). Total biomass stimulation was contributed by the overall increase in root, stem, and leaf biomass along with an increase in leaf area, leaf number, tiller number, and spike number (Table 3; Supplementary Table S2). At the final harvest, eCO_2_-grown plants had 35% (*P*<0.001) more biomass and 30% higher grain yield (*P*=0.001) than aCO_2_-grown plants under control conditions (Fig. 5; Table 3; Supplementary Table S2). The increase in grain yield of control plants under eCO_2_ was due to an increased number of tillers and consequently ears (+22%, *P*<0.001), while the main shoot grain yield was not stimulated (Supplementary Table S2).

**Table 3.** Summary of statistics for plant dry mass (DM) and morphological parameters

Time point	Parameter (mean per plant)	Main effects		Interaction
		CO_2_	HS	CO_2_×HS
13 WAP (T1)	Tiller number	**		
	Leaf number	***		
	Leaf area (cm^2^)	***		
	Ear number	***		
	Ear DM (g)	***		
	Leaf DM (g)	***		
	Stem DM (g)	***		
	Roots DM (g)	NS		
	Shoot DM (g)	***		
	Total DM (g)	***		
17 WAP (T2)	Tiller number	***	***	***
	Leaf number	***	***	***
	Leaf area (cm^2^)	***	NS	**
	Ear number	***	***	***
	Ear DM (g)	***	***	NS
	Leaf DM (g)	***	***	***
	Stem DM (g)	***	***	***
	Roots DM (g)	***	**	*
	Shoot DM (g)	***	***	NS
	Total DM (g)	***	***	NS
25 WAP (T3)	Ear number	***	***	*
	Ear DM (g)	***	***	NS
	Roots DM (g)	NS	***	NS
	Shoot DM (g)	***	***	**
	Total DM (g)	***	NS	NS
	Main stem grain yield (g)	NS	***	NS
	Grain yield (g)	***	***	NS
	Grain number	**	***	NS
	Grains per ear	NS	***	NS
	Grain size (mg per grain)	**	***	NS
	Harvest index	**	***	*

Summary of statistical analysis using two-way ANOVA for the effects of elevated CO_2_ and heat stress (HS) on biomass and morphological parameters for plants harvested at various time points (*n*=9–10). Significance levels are *** *P* <0.001; **P<0.01; *P<0.05; NS, *P*>0.05

HS reduced the biomass of aCO_2_ plants (–30%, *P*<0.001) more than eCO_2_ plants (–10%, *P*=0.09) harvested at 17 WAP following the HS (Fig. 5; Supplementary Table S2). By the final harvest, HS plants recovered and had similar biomass relative to control plants grown under both aCO_2_ and eCO_2_. This recovery in biomass was driven by the HS-induced stimulation of additional late tillers and consequently new ears (Fig. 5).

**Fig. 6:**
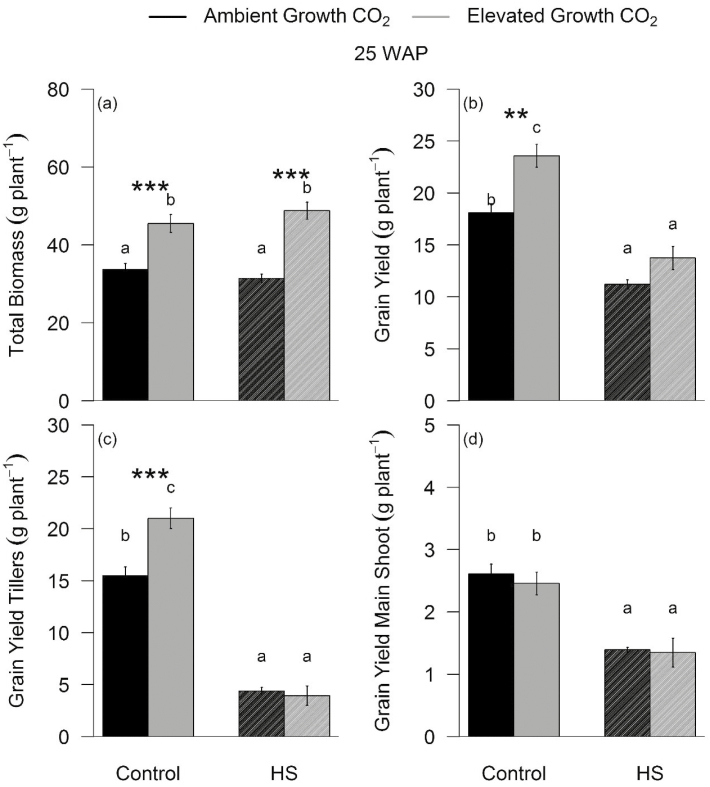
Response of plant total biomass and grain yield to elevated CO_2_ and heat stress (HS) at the final harvest. Bar plot of means ±SE for total biomass (a), grain yield (b), grain yield of tillers (c), and grain yield of the main shoot (d) using two-way ANOVA measured in ambient (black) and elevated (grey) CO_2_-grown plants exposed (HS) or not exposed (Control) to a 5 d HS. Bars sharing the same letter in the individual panels are not significantly different according to Tukey’s HSD test at the 5% level. Values are means ±SE (*n*=9–10). Statistical significance levels (*t*-test) for eCO_2_ effect are shown: *P<0.05; **P<0.01: ***P<0.001.

Despite the recovery in biomass, the grain yield was similarly reduced by HS in both aCO_2_- (–38%, *P*<0.001) and eCO_2_- (–41%, *P*<0.001) grown plants due to grain abortion in old ears and insufficient grain filling in new ears (Fig. 6a, b; Supplementary Table S2). HS reduced grain yield of tillers (–77%, *P*<0.001) more than the main shoot (–45%, *P*<0.001), which developed earlier. In addition, HS reduced grain yield of tillers in plants grown at aCO_2_ (–71 % *P*<0.001) less than in those grown at eCO_2_ (–81%, *P*<0.001) due to their higher tiller number (Fig. 6c, d). This phenomenon is well recognized as growth stimulation at eCO_2_ may limit grain yield due to trade-off between vegetative and reproductive components, including grains (Dias de Oliveira *et al.*, 2015). HS caused grain abortion, leading to empty ears without grains, or damaged and shrunken grains (Supplementary Fig. S5) evident from the reduction in grain per spike (–53%, *P*<0.001) and average grain weight (–25%, *P*<0.001) under both CO_2_ treatments.

## Discussion

Although field experiments are crucial to understand plant- and canopy-level responses to the environment, mechanistic and interactive analysis of climate change variables such as eCO_2_ and temperature remains challenging in the field. In this study, we investigated the interactive effects of eCO_2_ and HS on photosynthesis, biomass, and grain yield of Scout, a high-yielding modern wheat cultivar, under controlled environments. Wheat was grown at ambient or elevated CO_2_ and exposed to a 5 d HS at 50% anthesis. eCO_2_ stimulated photosynthesis, biomass, and grain yield, while HS reduced photosynthetic rates under aCO_2_ and eCO_2_. eCO_2_ improved the recovery of photosynthesis and biomass following HS, but the HS-induced reduction in grain yield was similar under both CO_2_ treatments due to grain abortion and inadequate grain filling. Our study demonstrated the interactive effects between eCO_2_ and HS, providing insights into the mechanisms underlying the interactions, and identified a major discrepancy between the response of wheat photosynthesis and biomass versus grain yield to eCO_2_×HS. The novelty of this study is in linking photosynthesis, biomass, and grain yield responses to the interactive effects of future climate variables. Our results and modelled parameters will be useful in developing a mechanistic modelling approach based on photosynthesis and parametrization of crop models that can predict yield under future extreme climate. Our results suggest that grain filling and translocation to the grain at high temperature are key weaknesses in modern high-yielding wheat varieties. Moreover, plastic tillering in response to eCO_2_ and HS adversely impacted the final grain yield. These traits should be prioritized in current breeding programmes to sustain staple food production under future climate extremes.

### Elevated CO_2_ reduced photosynthetic electron transport capacity at high temperature

Elevated CO_2_ modulates the instantaneous temperature response of photosynthesis (Sage and Kubien, 2007; Ghannoum *et al.*, 2010). Growth at eCO_2_ slowed down the rate of increase in *V*_cmax_ and accentuated the decrease in *J*_max_ above *T*_opt_ in Scout (Fig. 2c), possibly due to reduced Rubisco activation and limitation in electron transport capacity at high temperature and eCO_2_ (Sage and Kubien, 2007). Contrary to our hypothesis that eCO_2_ will increase *T*_opt_ (Long, 1991), photosynthetic *T*_opt_ was similar under aCO_2_ and eCO_2_ (Table 2). Lower *V*_cmax_ and *J*_max_ at higher temperatures may have prevented the increase in *T*_opt_ under eCO_2_, because the temperature dependence of *J*_max_ determines the shift of optimal temperature of photosynthesis at eCO_2_ (Hikosaka *et al.*, 2006). Our results are consistent with a previous study by Alonso *et al.* (2008) where they found decreased *V*_cmax_ under eCO_2_. In other wheat studies, eCO_2_ increased *V*_cmax_ and *J*_max_ at supraoptimal temperatures, and reduced *J*_max_ at suboptimal temperatures (Alonso *et al.*, 2009). The discrepant *V*_cmax_ and *J*_max_ responses to our studies could be due to an unusual increase in *V*_cmax_ under eCO_2_ observed in the study by Alonso *et al.* (2009). Modelled values of *V*_cmax_ at 25 °C (150 μmol m^−2^ s^−1^ and 121 μmol m^−2^ s^−1^ at aCO_2_ and eCO_2_, respectively) are similar to *in vitro* (137 μmol m^−2^ s^−1^ and 123 μmol m^−2^ s^−1^ at aCO_2_ and eCO_2_, respectively) values measured in the current study and previously reported in wheat (117 μmol m^−2^ s^−1^) by Silva-Pérez *et al.* (2017). Modelled values of *E*_a_*V* (51 kJ and 38 kJ at aCO_2_ and eCO_2_, respectively) were lower than *E*_a_*V* (63 kJ) reported in the wheat study by Silva-Pérez *et al.* (2017).

### Elevated CO_2_ protected wheat photosynthesis by stimulating electron transport potential following HS

Generally, HS reduces net photosynthetic rates in wheat (Wang *et al.*, 2008), while the extent of the response depends on the cultivar (Sharma *et al.*, 2014). However, acclimation to long-term eCO_2_ can modulate the photosynthetic responses to HS during the vegetative or anthesis stage (Wahid *et al.*, 2007). In Scout, photosynthetic rates recovered following HS under eCO_2_ but not under aCO_2_ (Fig. 4a), indicating that HS transiently reduced photosynthesis without eliciting permanent damage to the photosynthetic apparatus of eCO_2_-grown plants. The recovery of photosynthesis under eCO_2_ was associated with the recovery of the electron transport rate (Fig. 4d) and photochemical efficiency (Fig. 4a), maintenance of *V*_cmax_ (Fig. 3b), and increased *J*_max_ (Fig. 3d) relative to non-HS, eCO_2_-grown plants, thus validating our hypothesis that eCO_2_ will protect photosynthesis via increased electron transport. Higher *J*_max_ may have protected the photosynthetic apparatus from HS damage by increasing electron sinks, and hence photochemical quenching (Sage and Kubien, 2007). Higher *J*_max_ is also associated with higher Rubisco activation (Perdomo *et al.*, 2017), which may have helped recovery of photosynthesis under eCO_2_.

In contrast, aCO_2_-grown Scout suffered permanent loss of photosynthesis and photochemical efficiency (*F*_v_/*F*_m_) after HS. Reduced *F*_v_/*F*_m_ is a sign of stress (Sharkova, 2001; Haque *et al.*, 2014) and indicates lower quantum efficiency of PSII (Baker, 2008). Damage to the photosynthetic apparatus was also evident from the reduction in *V*_cmax_ at aCO_2_ (Fig. 3b), although *J*_max_ was not affected by HS (Fig. 3d). Consequently, the *J*_max_/*V*_cmax_ ratio was equally increased by HS in both CO_2_ treatments, suggesting increased resource allocation to RuBP regeneration or electron transport (Hikosaka *et al.*, 2006) in response to HS irrespective of growth CO_2_. An enhanced *J*_max_/*V*_cmax_ ratio by exposure to HS may potentially play a role in avoiding photoinhibition (Walker *et al.*, 2014).

In line with our results, photosynthesis and *F*_v_/*F*_m_ were inhibited by HS (3 d at 40 °C) applied after anthesis in two wheat cultivars grown at aCO_2_ but not at eCO_2_ (Shanmugam *et al.*, 2013). Protection from HS damage of photosynthesis as a result of improved photochemical quenching or electron transport appears to be a universal mechanism in crops exposed to eCO_2_. In tomato, HS (42 °C) reduced *A*_sat_ (–57%), *V*_cmax_, and *J*_max_ (–45%) under aCO_2_, while eCO_2_ increased *A*_sat_ (+96%), *V*_cmax_, and *J*_max_ after 24 h of recovery from HS (Pan *et al.*, 2018). In Arabidopsis, photosynthesis and chlorophyll fluorescence were less inhibited by HS (38 °C) in eCO_2_ than in aCO_2_ 8 d after recovery (Zinta *et al.*, 2014). The study concluded that eCO_2_ mitigated HS stress impacts through up-regulation of antioxidant defence metabolism and reduced photorespiration, resulting in lowered oxidative pressure (Zinta *et al.*, 2014). In other studies investigating the interactive effects of eCO_2_ and HS (reviewed by Wang *et al.*, 2011), eCO_2_ enhanced the thermal tolerance of photosynthesis in both cool- and warm-season species, indicating that the mitigating effects of CO_2_ were independent of the plant habitat (Hogan *et al.*, 1991; Wang *et al.*, 2008).

### Following HS, plant biomass recovered in all plants due to late tillering, while grain yield declined even under eCO_2_

Despite the initial negative impacts of HS on plant growth in Scout, total plant biomass recovered at maturity, and this was associated with positive source (photosynthesis) and sink (tiller) responses. As discussed earlier, HS caused irreversible photosynthetic damage at aCO_2_, while growth at eCO_2_ mitigated the negative impact of HS on photosynthesis. Moreover, the biomass of HS plants recovered under both CO_2_ treatments due to late tiller and ear development (Bányai *et al.*, 2014). When grain development is stalled under certain conditions (e.g. HS), the crop develops new grains by producing additional late tillers. This is considered a non-harmful acclimation response to HS which creates additional sinks. Hence, grain abortion due to HS was compensated by the production of additional late tillers contributing to the recovery in biomass at the final harvest. An equal decrease in biomass under aCO_2_ and eCO_2_ following exposure to HS has been reported in a study using the C_3_ crop *Sinapis alba* (white mustard), which also concluded that interactive effects of CO_2_ and HS depend on species, magnitude of HS, and growth conditions (Coleman *et al.*, 1991). In cases where HS causes persistent reduction in biomass at aCO_2_, eCO_2_ often alleviates the negative impacts of HS (Zinta *et al.*, 2014). It is worth noting that the development of additional late ears and tillers following HS is expected to increase sinks for the translocation of assimilates. Greater sink strength may partly explain photosynthetic recovery in HS plants (Paul and Foyer, 2001). However, photosynthesis recovered in eCO_2_ plants only, while late tillering was observed under both CO_2_ treatments. Similarly, in wheat grown using growth chambers and exposed to moderate HS (32 °C) after anthesis, grain yield decreased under both ambient and elevated CO_2_ (Zhang *et al.*, 2018). Althogh Scout biomass recovered in all plants exposed to HS, grain yield was equally reduced in both CO_2_ treatments due to grain abortion in the old ears and insufficient time for grain filling in the new ears. In response to HS, some ears had completely lost grains, and ears with developing grains could not fill, leading to shrunken and damaged grains (Supplementary Fig. S5), and hence a significant loss of grain yield consistent with previous studies (Stone and Nicolas, 1996, 1998; Spiertz *et al.*, 2006; Prasad and Djanaguiraman, 2014).

Observed HS damage to grain yield was higher in tillers than in the main shoot (Fig. 6c, d) due to high senstivity of wheat at heading and anthesis stages (Prasad and Djanaguiraman, 2014). When HS was applied, the main shoots may have been past anthesis while tillers were in the heading or anthesis stages, and thus more exposed to HS impacts (Prasad and Djanaguiraman, 2014). FACE studies (Fitzgerald *et al.*, 2016; Macabuhay *et al.*, 2018) in wheat involving interactive effects eCO_2_ and HS found that eCO_2_ can buffer against heat waves, and eCO_2_ may moderate some effects of HS in wheat depending on seasonal conditions and HS timing.

In conclusion, eCO_2_ stimulated photosynthesis, biomass, and grain yield in a modern, high-yielding wheat variety. In non-HS plants, photosynthetic stimulation by eCO_2_ was observed despite reduction of *V*_cmax_ at all temperatures and *J*_max_ at higher temperatures. In heat-shocked plants, eCO_2_ stimulated *J*_max_ and maintained photochemical efficiency, hence providing photosynthetic protection against HS damage. Consequently, HS reduced photosynthesis under aCO_2_ more than under eCO_2_. Plant biomass completely recovered from HS under both CO_2_ treatments due to the development of additional late tillers and ears; yet these did not fully develop and fill grains. Therefore, HS applied at anthesis equally reduced grain yield under aCO_2_ and eCO_2_ due to grain abortion. In the field, late tillers would not necessarily produce higher grain yield either, because plants will run out of soil water and there is not enough time for grain filling. The current study demonstrates the interactive impacts of eCO_2_ and severe HS applied at 50% anthesis on wheat yield. HS can occur over a wide window from booting to late grain-filling stage, thus affecting yield in variable ways and limiting the generalization of our results. Nonetheless, our study provides insights into the interactive effects of eCO_2_ and HS on the thermal responses of wheat photosynthesis which apply over a wide range of scenarios, and hence can form the basis for crop models to incorporate the interactive effects of eCO_2_ and HS.

## Supplementary data

Supplementary data are available at *JXB* online.

**Table S1**. Response of leaf gas exchange parameters to elevated CO_2_ and heat stress.

**Table S2**. Response of plant dry mass and morphological parameters to elevated CO_2_ and heat stress.

**Table S3**. Temperature response of mesophyll conductance in Scout.

**Fig. S1**. Glasshouse growth conditions and heat stress cycle.

**Fig. S2**. Radiation over time during the experiment.

**Fig. S3**. Experimental design depicting plant growth plotted over time.

**Fig. S4**. Temperature response of spot gas exchange parameters.

**Fig. S5**. Response of grain size and morphology to heat stress.

erz386_suppl_Supplementary_Tables_S1-S3_Figures_S1-S5
