# Supplementary material for: Elevated CO2 alleviates the negative impact of heat stress on wheat physiology but not on grain yield
Source: J Exp Bot. 2019 Aug 24;70(21):6447–59. doi: 10.1093/jxb/erz386 (PMC6859723; doi:10.1093/jxb/erz386)
Supplement: erz386_suppl_Supplementary_Tables_S1-S3_Figures_S1-S5 [file erz386_suppl_supplementary_tables_s1-s3_figures_s1-s5.pdf]

Table S1 Response of leaf gas exchange parameters to elevated CO_2 and heat stress: Summary of leaf gas exchange parameters measured at different time points for wheat cultivar Scout grown at ambient CO_2 (aCO_2) or elevated CO_2 (eCO_2) and exposed (H) or not exposed (control) to 5-day heat stress (HS) at the anthesis stage. Values are means ± SE (n= 9-10).

Parameter	Growth	Measurement		T1 (13WAP)	HS Cycle (15WAP)			T2 (17WAP)	
		Temp	CO_2	Control	Before (Control)	During (HS)	After (HS)	Recovery (HS)	Control
A (µmol $m^{-2}s^{-1}$)	aCO_2	25	400	31 ± 0.3	28.3 ± 0.8	23.7 ± 0.8	14.2 ± 1.2	13.4 ± 2.6	23.1 ± 1.2
		25	650	44.2 ± 0.7	38.1 ± 1.1	30 ± 1.1	20.2 ± 1.4	19.1 ± 3.3	31.8 ± 1.9
		35	400	26.4 ± 0.4	25.4 ± 0.7	21.7 ± 0.8	15.6 ± 1.1	11.2 ± 2	18.6 ± 1.9
		35	650	42.3 ± 0.8	39.8 ± 0.8	33.1 ± 1.1	25.9 ± 1.8	19.8 ± 2.9	28.7 ± 2.6
	eCO_2	25	400	27.4 ± 0.7	27.1 ± 0.8	28 ± 1.1	18.7 ± 1	22.9 ± 1	22 ± 0.9
		25	650	38.6 ± 1.3	40.5 ± 1.1	35.4 ± 1.2	27.1 ± 1.4	34.6 ± 1.8	31.6 ± 1
		35	400	23.3 ± 0.8	24 ± 0.7	24.9 ± 1.2	19.2 ± 1.1	17.4 ± 2.1	13.6 ± 1.9
		35	650	36.8 ± 0.9	38.9 ± 1	38.5 ± 1.8	30 ± 2.2	29.8 ± 3.2	22.7 ± 4
Rd (µmol $m^{-2}s^{-1}$)	aCO_2	25	400	-2.4 ± 0.2	-1.8 ± 0.1	-2.5 ± 0.1	-2.4 ± 0.1	-1.9 ± 0.3	-1.8 ± 0.1
		35	400	-4.0 ± 0.2	-2.9 ± 0.2	-3.7 ± 0.1	-3 ± 0.2	-2.6 ± 0.2	-2.8 ± 0.2
	eCO_2	25	400	-1.9 ± 0.2	-2.6 ± 0.2	-2.3 ± 0.1	-2.9 ± 0.1	-2.3 ± 0.2	-2.1 ± 0.1
		35	400	-3.1 ± 0.3	-3.7 ± 0.1	-3.9 ± 0.2	-4.5 ± 0.2		-3.3 ± 0.2
g_s (mol $m^{-2}s^{-1}$)	aCO_2	25	400	0.42 ± 0.02	0.41 ± 0.03	0.54 ± 0.02	0.22 ± 0.02	0.26 ± 0.03	0.38 ± 0.01
		25	650	0.42 ± 0.02	0.4 ± 0.03	0.54 ± 0.02	0.23 ± 0.02	0.25 ± 0.02	0.41 ± 0.02
		35	400	0.29 ± 0.01	0.4 ± 0.03	0.49 ± 0.03	0.41 ± 0.03	0.26 ± 0.03	0.23 ± 0.03
		35	650	0.29 ± 0.01	0.39 ± 0.02	0.47 ± 0.03	0.44 ± 0.04	0.25 ± 0.02	0.23 ± 0.03
	eCO_2	25	400	0.4 ± 0.02	0.47 ± 0.03	0.66 ± 0.04	0.31 ± 0.02	0.39 ± 0.04	0.41 ± 0.04
		25	650	0.38 ± 0.02	0.49 ± 0.02	0.63 ± 0.03	0.3 ± 0.02	0.41 ± 0.02	0.43 ± 0.03
		35	400	0.26 ± 0.02	0.43 ± 0.02	0.54 ± 0.03	0.36 ± 0.02	0.24 ± 0.04	0.19 ± 0.04
		35	650	0.27 ± 0.02	0.43 ± 0.02	0.53 ± 0.03	0.35 ± 0.04	0.24 ± 0.04	0.19 ± 0.05
PWUE (A_{sat}/g_s) (µmol mol^{-1})	aCO_2	25	400	74 ± 3	72 ± 4	44 ± 2	66 ± 3	51 ± 5	61 ± 2
		25	650	107 ± 6	98 ± 6	56 ± 3	89 ± 3	74 ± 7	77 ± 4
		35	400	92 ± 4	65 ± 3	46 ± 3	39 ± 3	43 ± 6	83 ± 10
		35	650	146 ± 6	105 ± 5	72 ± 5	61 ± 4	78 ± 9	127 ± 7
	eCO_2	25	400	69 ± 2	58 ± 2	43 ± 2	62 ± 4	61 ± 6	55 ± 4
		25	650	101 ± 3	84 ± 3	57 ± 3	95 ± 7	85 ± 3	75 ± 5
		35	400	91 ± 6	57 ± 2	48 ± 3	54 ± 4	75 ± 6	75 ± 11
		35	650	139 ± 10	93 ± 4	75 ± 5	99 ± 15	132 ± 14	134 ± 25
Fv'/Fm'	aCO_2	25	400	0.48 ± 0.02	0.5 ± 0.01	0.47 ± 0.01	0.41 ± 0.01	0.33 ± 0.04	0.46 ± 0.02
		35	400	0.42 ± 0.01	0.45 ± 0.01	0.42 ± 0.02	0.35 ± 0.01	0.31 ± 0.04	0.4 ± 0.01
	eCO_2	25	400	0.48 ± 0.03	0.52 ± 0.01	0.47 ± 0.01	0.45 ± 0.01	0.46 ± 0.01	0.47 ± 0.01
		35	400	0.41 ± 0.01	0.47 ± 0.01	0.41 ± 0.01	0.41 ± 0.01	0.42 ± 0.01	0.37 ± 0.04
Fv/Fm	aCO_2	25	400	0.82 ± 0.01	0.8 ± 0.01	0.75 ± 0.01	0.75 ± 0.01	0.73 ± 0.02	0.76 ± 0.01
		35	400	0.79 ± 0.01	0.8 ± 0	0.73 ± 0.02	0.72 ± 0.01	0.72 ± 0.04	0.78 ± 0.01
	eCO_2	25	400	0.8 ± 0.02	0.82 ± 0	0.79 ± 0.01	0.76 ± 0.01	0.78 ± 0.01	0.78 ± 0.01
		35	400	0.79 ± 0.01	0.81 ± 0	0.75 ± 0.01	0.76 ± 0.01		0.74 ± 0.04

Table S2 Response of plant dry mass (DM) and morphological parameters to elevated CO_2 and heat stress: Summary of plant biomass and morphological parameters measured at different time points for what cultivar Scout grown at ambient CO_2 (aCO_2) or elevated CO_2 (eCO_2), with some plants exposed to heat stress (HS) at the anthesis stage. Values are means ± SE (n= 9-10).

Parameter (Mean plant^{-1})	Time	T1(13WAP)	T2 (Anthesis, 17 WAP)		T3 (Maturity, 25	
	Heat Stress	Control	Control	HS	Control	HS
	Growth CO_2					
Tiller Number	aCO_2	9.2 ± 0.6	10.9 ± 0.5	16.3 ± 0.7		
	eCO_2	12.9 ± 0.7	12.3 ± 0.8	28.3 ± 1.6		
Leaf Area (cm^2)	aCO_2	338 ± 27	338 ± 40	296 ± 47		
	eCO_2	680 ± 59	441 ± 58	650 ± 55		
Leaf Number (n)	aCO_2	33.8 ± 2.6	26.9 ± 0.8	37 ± 3.1		
	eCO_2	49.5 ± 2.8	27.6 ± 2.2	66.7 ± 4.4		
Leaf Size (cm^2)	aCO_2	27 ± 7	32 ± 5	38 ± 5		
	eCO_2	24 ± 2	24 ± 3	42 ± 5		
Leaf Mass Area	aCO_2	58 ± 1	56 ± 2	68 ± 1		
	eCO_2	52 ± 2	48 ± 3	60 ± 1		
Leaf DM (g)	aCO_2	1.9 ± 0.1	1.8 ± 0.1	2.0 ± 0.3		
	eCO_2	3.5 ± 0.2	1.8 ± 0.1	3.9 ± 0.3		
Stem DM (g)	aCO_2	3.9 ± 0.1	9.6 ± 0.3	9.3 ± 0.7		
	eCO_2	8.5 ± 0.6	11.4 ± 0.8	17.1 ± 0.7		
Root DM (g)	aCO_2	1.4 ± 0.2	1.6 ± 0.1	1.9 ± 0.2	0.6 ± 0.1	2 ± 0.3
	eCO_2	2.0 ± 0.2	2.4 ± 0.4	3.8 ± 0.8	0.6 ± 0.1	1.2 ±
Shoot DM (g)	aCO_2	8.1 ± 0.3	32.9 ± 1.3	22.1 ± 0.8	9.3 ± 0.4	10.3 ±
	eCO_2	16.8 ± 1.1	41.2 ± 1.9	35.5 ± 0.8	12.7 ± 0.7	18.9 ±
Total DM (g)	aCO_2	9.5 ± 0.4	34.5 ± 1.5	24.1 ± 1.0	33.7 ± 1.5	31.4 ±
	eCO_2	18.8 ± 1.3	43.6 ± 2.2	39.3 ± 0.9	45.5 ± 2.2	48.8 ±
Ear Number	aCO_2				10.2 ± 0.3	16.7 ±
	eCO_2				12.5 ± 0.4	22.6 ±
Grains Per Ear	aCO_2				41 ± 1	21 ± 1
	eCO_2				41 ± 1	17 ± 1
Total Grain Number	aCO_2				415 ± 21	352 ±
	eCO_2				511 ± 20	384 ±
Single Grain Weight	aCO_2				44 ± 1	32 ± 1
	eCO_2				46 ± 1	35 ± 1
Grain yield (g)	aCO_2				18.1 ± 0.9	11.2 ±
	eCO_2				23.5 ± 1.1	13.7 ±
Harvest Index	aCO_2				0.536 ±	0.357 ±
	eCO_2				0.518 ±	0.281 ±

Table S3 Temperature response of mesophyll conductance (g_m) in Scout: Summary of mesophyll conductance measured at five leaf temperatures using concurrent gas exchange and stable carbon isotope measurements. Values are means ± SE (n=4)

Leaf Temperature (°C)	Mesophyll Conductance (g_m, mol $m^{-2}s^{-1}$)
**15**	0.232 ± 0.005
**20**	0.286 ± 0.01
**25**	0.309 ± 0.008
**30**	0.279 ± 0.007
**35**	0.292 ± 0.01

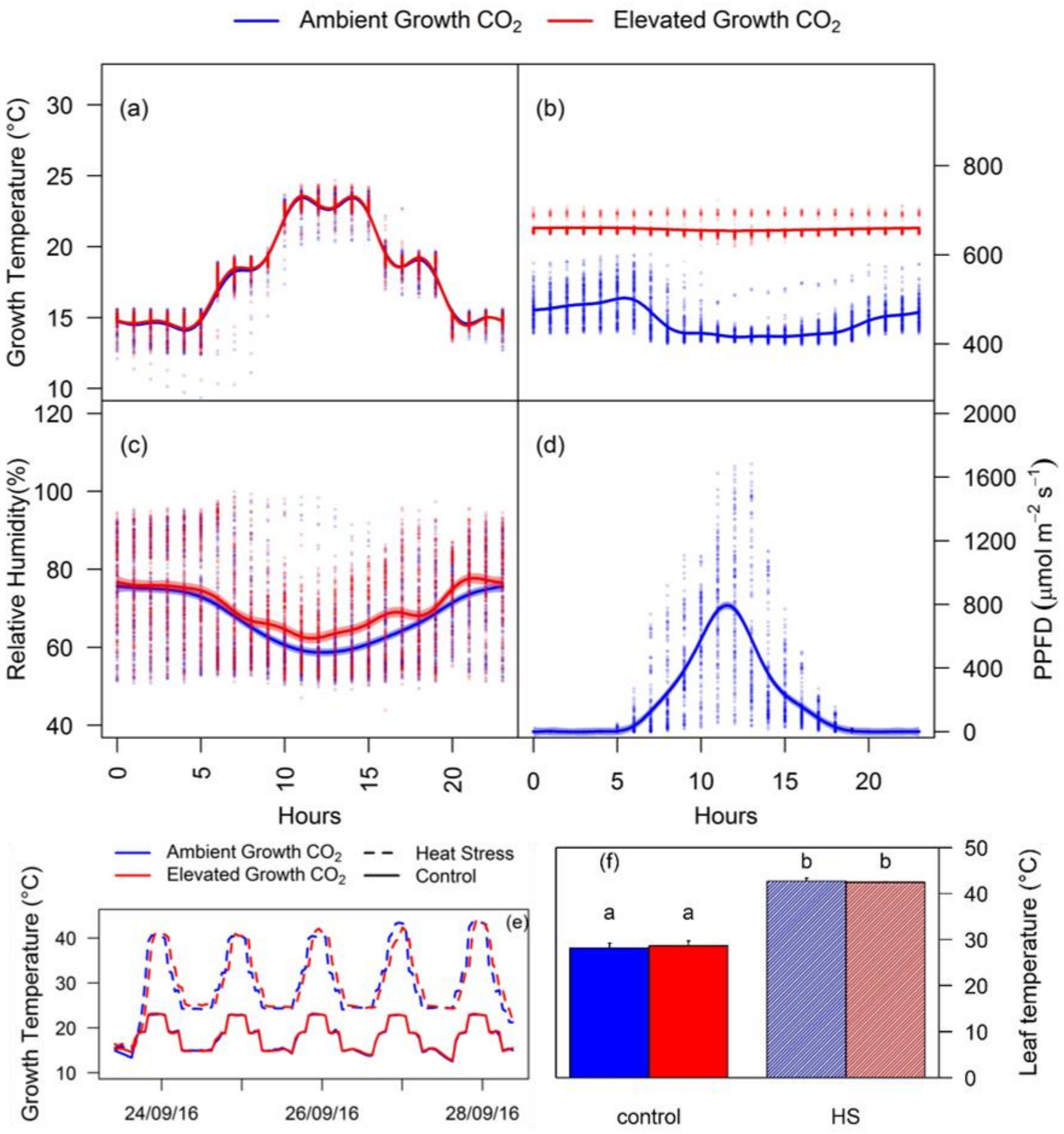

Figure S1 Glasshouse growth conditions and heat stress cycle

Daily averages of growth temperature (a), CO_2 (b), relative humidity (c) and PPFD (d). In panels, a, b, c and d solid lines represent the growth averages, while the faint data points show recorded observations. Lower panel (e) illustrates the 5-day heat stress cycle at the anthesis stage. Growth air temperatures recorded in control and heat stress chambers during heat stress are depicted using solid and dotted lines, respectively. Bar plot of means for leaf temperature (f) of 2-way analysis of variance (anova). Values represent means ± standard error (n=5). Bars sharing the same letter in the individual panel are not significantly different according to Tukey's HSD test at the 5% level. Ambient and elevated CO_2 treatments are depicted in blue and red, respectively.

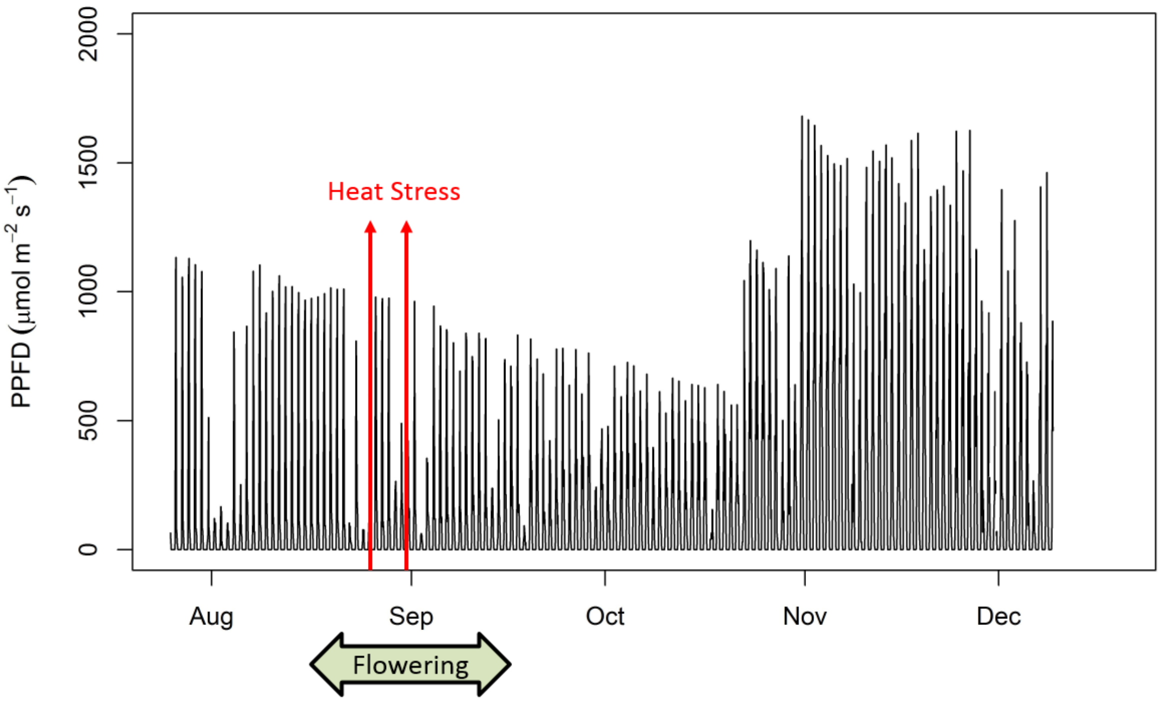

Figure S2 Radiation over time

Radiation over time depicting the radiation load during the experiment highlighted for anthesis and heat stress.

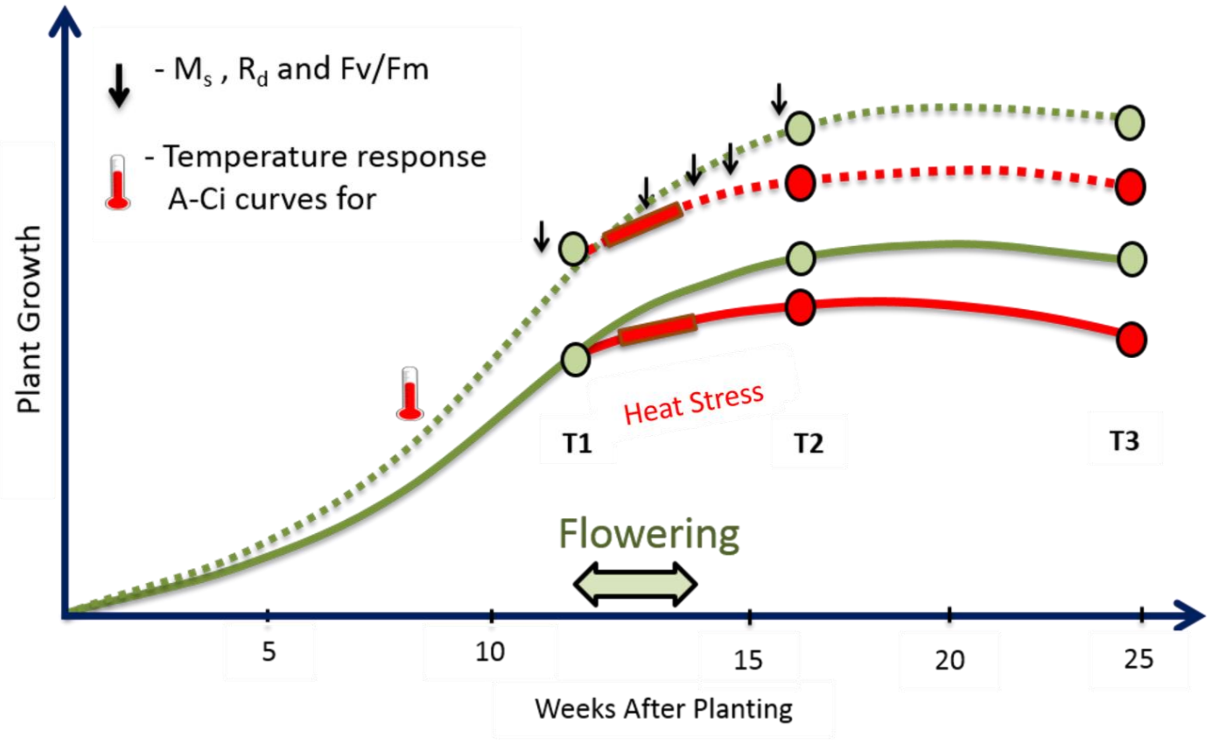

Figure S3 Experimental design depicting plant growth plotted over time showing harvesting at 3-time points (T1, T2 and T3) across the wheat life cycle till maturity, timing of heat stress and measurements

The circles represent harvest of 10 plants at corresponding time point. Green circles on the green solid and dotted lines represent control plants grown at ambient and elevated CO_2 respectively. Red rectangles point to timing and duration of the heat stress (HS) at anthesis stage. The red circles on red solid and dotted lines represent plants subjected to heat stress and grown at ambient and elevated CO_2 respectively. Thermometer symbol represents timing of temperature response measurements.

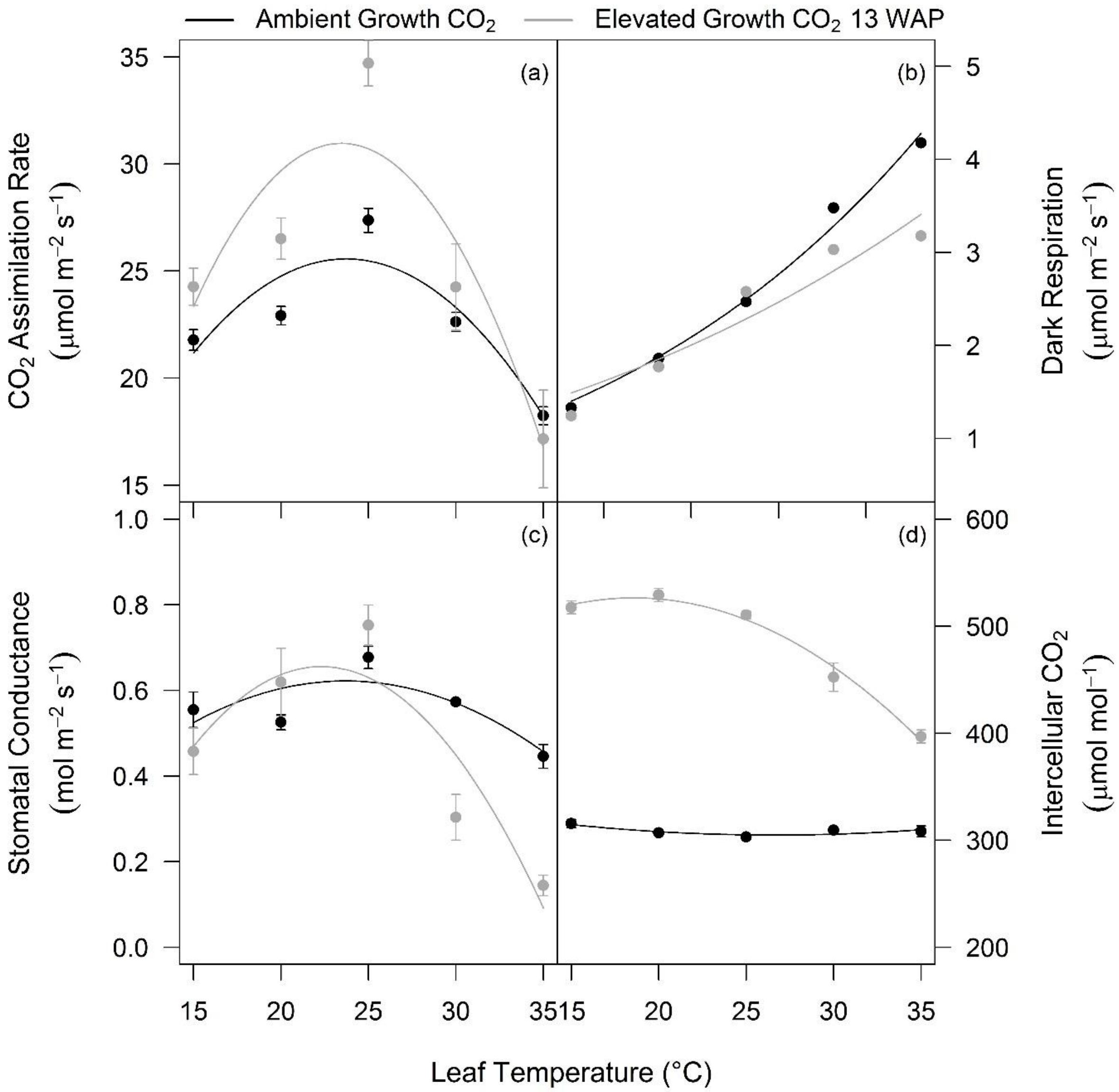

Figure S4 Temperature response of spot gas exchange parameters measured thirteen weeks after planting (WAP)

CO_2 assimilation rate (a), dark respiration (b), stomatal conductance (c) and intercellular CO_2 (d) measured at growth CO_2 and five leaf temperatures (15, 20, 25, 30 and 35 ^{o}C) in wheat cultivar Scout. Values are mean ± SE (n=6). Ambient and elevated CO_2 grown plants are depicted in black and grey, respectively.

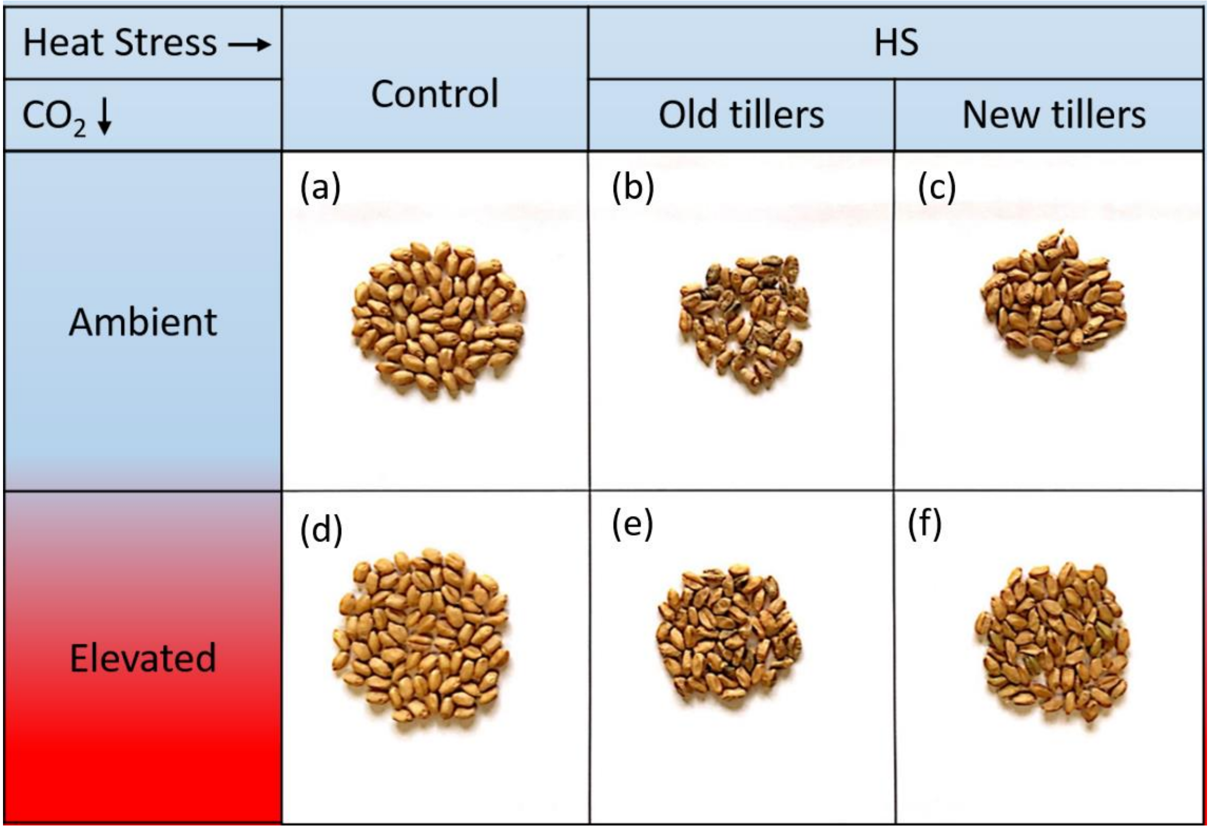

Figure S5 Response of grain size and morphology to heat stress at the final harvest

Heat stress effect on grain size and morphology from old tillers and additional late tillers developed after HS under ambient (a, b, c) and elevated CO_2 (d, e, f).
